# The effects of suppressing the biological stress systems on social threat-assessment following acute stress

**DOI:** 10.1007/s00213-020-05591-z

**Published:** 2020-06-29

**Authors:** Nida Ali, Cory Cooperman, Jonas P. Nitschke, Mark W. Baldwin, Jens C. Pruessner

**Affiliations:** 1 Department of Psychology, McGill University, 2001 Avenue McGill College, Montreal, H3A 1G1 Canada; 2 Faculty of Medicine, McGill Centre for Studies in Aging, McGill University, Montreal, Canada; 3 Department of Clinical and Health Psychology, University of Vienna, Vienna, Austria; 4 Department of Psychology, University of Konstanz, Konstanz, Germany

**Keywords:** Stress, Threat assessment, Contextual effects, HPA axis, SNS, Cortisol, Salivary alpha amylase, Pharmacological challenge, Cognition

## Abstract

**Rationale:**

Stress is associated with increased sensitivity to threat. Previous investigations examining how stress affects threat processing have largely focused on biomarker responses associated with either the sympathetic-nervous-system (SNS) or the hypothalamus-pituitary-adrenal (HPA) axis.

**Objectives:**

We pharmacologically suppressed activations of SNS, HPA, or both, prior to stress and investigated how each stress system modulates social threat assessment.

**Methods:**

One hundred sixty-one healthy men and women were randomized in a between-subject design, to one of four pharmacological or placebo conditions: dexamethasone–placebo, placebo–propranolol, dexamethasone–propranolol, or placebo–placebo. Participants provided threat assessments for angry and neutral human faces on a baseline day, and immediately after stress induction on a testing day.

**Results:**

With both systems responding normally to stress (placebo–placebo), threat assessment was higher for neutral faces compared with angry. Compared with placebo, SNS suppression resulted in increased threat assessment for angry faces. HPA suppression resulted in decreased threat assessment for neutral and angry faces. When both systems were suppressed, there was an increase in threat assessment for angry faces, and no difference from placebo for neutral.

**Conclusion:**

Our findings demonstrated that when intact, the biological stress systems adaptively support organisms during stress by focusing attention towards specific stimuli that are relevant to the threat. Dysregulations of the stress systems result in important system specific consequences on threat evaluation, such that suppression of either stress system alone resulted in reduced threat assessment for contextually relevant threatening stimuli, whereas when both systems were suppressed, individuals appear indiscriminately attentive to all potential threats in the environment, resulting in increased threat processing of both contextually relevant and irrelevant stimuli. Given that stress-related psychopathologies have been associated with dysregulations of the stress systems and biased responses to social threat, a systematic understanding of the mechanisms that underlie how stress systems modulate social threat assessment is needed, and can provide important insights into the cognitive processes that are involved in the development and maintenance of stress-related psychopathologies.

**Electronic supplementary material:**

The online version of this article (10.1007/s00213-020-05591-z) contains supplementary material, which is available to authorized users.

## Introduction

To successfully navigate in an information rich world, we need to be able to efficiently process a constant influx of stimuli and discriminately attend to only the relevant information. This ability becomes even more critical when we are confronted with threat and have to quickly and accurately identify it, and mount appropriate responses to deal with it. Thus, given the significant impact that threat assessment has on our survival and wellbeing, it is crucial to understand how stress affects the ability to correctly identify and assess threat.

When confronted with acute stress, the body’s physiological responses are activated to help organisms adaptively cope with the stressor. This encompasses activation and coordination among the sympathetic-nervous-system (SNS) and the hypothalamic-pituitary-adrenal (HPA) axis. SNS activation results in the release of catecholamines—adrenaline (A) and noradrenaline (NA) by the locus coeruleus (LC)—and the secretion of salivary Alpha-Amylase (sAA), while activation of the HPA axis triggers the release of the glucocorticoid (GC) hormone, cortisol, from the adrenal-cortex. Importantly, both catecholamines and GC have been shown to modulate threat processing in limbic regions, particularly the hippocampus and amygdala (Herman et al. 2005), along with other brain areas involved in stress appraisal, such as the prefrontal cortex (Roozendaal 2002). While numerous studies have investigated the individual effects of either SNS or HPA axis activation on attentional processes, only a few have examined how they affect threat processing during stress (Abercrombie et al. 2005; Kukolja et al. 2008), and fewer still have considered the activation (and contribution) of the stress system as a whole. To this end, here we used pharmacological challenges to systematically suppress activations of SNS, HPA, or both, prior to acute stress, to investigate how each stress system, independently activated, modulates social threat assessment.

During stress, behavioral and neural responses switch from primarily goal oriented, to reflexive and/or stimulus driven (Corbetta and Shulman 2002; Schwabe and Wolf 2009; Wirz et al. 2017, 2018). In fact, even in the absence of stress, when confronted with stimuli that are typically associated with threat (e.g., pictures of fearful faces), the attention system reflexively orients towards them indicating heightened systemic sensitivity, or vigilance, towards cues that are typically associated with threat (Vuilleumier 2002). This represents an evolutionarily adaptive mechanism that serves to alert the organism towards threat and allows it to behaviorally respond accordingly (Vuilleumier 2002; Putman and Roelofs 2011). Furthermore, this ability to identify and adaptively respond to relevant threatening stimuli is crucial for successful emotion regulation under stress (Gross 2002; Rueda et al. 2004). However, adaptive physiological, emotional, and behavioral responses to stress are contingent not only on the ability to attend to the source of stress but also to appropriately interpret the stimulus as threatening (Ellenbogen et al. 2006; Hermans et al. 2011). Here, only a few studies have investigated how neural and/or biomarker responses associated with stress modulate appraisal of emotional stimuli, with mixed results. For example, Abercrombie et al. (2005) found that higher cortisol was associated with increased arousal ratings for neutral faces. Likewise, Chen et al. (2014) found that ambiguous facial expressions were more likely to be miscategorized as fearful, after stress. In contrast, other studies have found that stress results in increased amygdalar hyper-vigilance for emotional stimuli, with no differences between positively or negatively valenced emotional stimuli (Kukolja et al. 2008; van Marle et al. 2009). Thus, while these studies have shown that stress has important effects on threat appraisal, they did not demonstrate how the stress systems were involved in modulating responses to threat.

To address these gaps in the literature, we used pharmacological challenges (propranolol and dexamethasone administration) to suppress the activity of the SNS and HPA axis to examine their separate and combined effects on threat evaluation during stress. Pilot studies by our group have demonstrated the efficacy of combining propranolol and dexamethasone challenges with stress induction in healthy men (Andrews et al. 2012; Andrews and Pruessner 2013). Propranolol is a beta-adrenergic receptor blocker commonly used to treat hypertension, anxiety, and tremors (Fraundorfer et al. 1994). It acts within the limbic system centrally, and on beta-adrenergic receptors peripherally, and has been shown to reduce cardiac reactivity, and sAA secretion, in acute stress paradigms (Benschop et al. 1996; Andrews and Pruessner 2013). Dexamethasone is a potent synthetic glucocorticoid that binds primarily to the GC receptors in the periphery and the pituitary, resulting in an almost complete suppression of ACTH secretion, which lasts into the morning following DEX administration the evening before. ACTH suppression in turn leads to the absence of cortisol production and secretion from the adrenal glands (de Kloet et al. 1974; Karssen et al. 2005). Based on previous literature, the following hypotheses were tested: (1) under placebo, participants would show increased threat assessment (from baseline) for emotional faces after stress. (2) Given the role of SNS in threat processing (Aston-Jones et al. 1994), compared to placebo, SNS suppression would result in decreased threat assessment for emotional stimuli. (3) Likewise, since cortisol administration is associated with increased attention to threat (Putman and Roelofs 2011), HPA axis suppression would result in decreased assessment of emotional threat. (4) In the combined dexamethasone-propranolol condition, threat assessment of emotional stimuli would be similar to placebo (Ali et al. 2017).

## Methods

The data presented in this study are part of a large project whose aim was to systematically manipulate the biological stress systems during acute stress, in order to investigate the interrelationships among them and the downstream effects on various facets that are involved in/impacted by stress. These include measures of subjective stress and affect, as well as cognitive processes including attention, threat appraisal, and memory. Data collection for the project occurred in two waves. Wave 1 (*n* = 80) occurred from November 2012–June 2013. A subset of the data from wave 1 (*n* = 44), investigating the efficacy of the combined propranolol and dexamethasone administration on stress biomarkers and psychological factors (mood, state-self-esteem, and subjective stress), has been published (Ali et al. 2017). Data collection for wave 2 (*n* = 81) occurred from May 2016–Feb 2018. The final dataset for the project thus comprises a combined sample size of *n* = 161 participants. While we describe sex differences in individual stress response system manipulation (either HPA or SNS suppression) and mood outcomes separately (Ali et al. 2020), the data presented in the current manuscript focus on the effects of manipulation of the biological stress response systems on threat assessments.

### Participants

Eighty men (mean age = 22.59, SD = 3.67, range = 19–32) and 81 women (mean age = 22.68, SD = 4.12, range = 18–35) were recruited via advertisements posted on the McGill University (Montreal, QC, Canada) online classifieds section. Individuals were excluded if they endorsed recreational drug use; consuming more than 10 alcoholic beverages per week; smoking more than 7 cigarettes per day; a history of, or current medical or psychiatric illness; using medications known to affect SNS or HPA-axis regulation. Women were tested during the luteal-phase of their menstrual cycle (17 to 28 days after onset of last menstruation), given that in this phase, the HPA axis stress response to laboratory stressors has been reported to be relatively comparable with those of men (Kirschbaum et al. 1999; Kudielka and Kirschbaum 2005). To establish the luteal-phase, women documented two menstrual cycles prior to being scheduled for the laboratory testing. All participants provided informed consent for the study, which was approved by the McGill University Faculty of Medicine Institutional Review Board.

Participants were randomly assigned to the following drug conditions (approx. 50% women): placebo (PLC; *n* = 41), dexamethasone (DEX; *n* = 40), propranolol (PROP; *n* = 40), and dexamethasone-propranolol (DP; *n* = 40). To ensure that both experimenters and participants remained blinded to the experimental manipulation, every participant received two pills, one to take at bedtime the night before testing (placebo or 2 mg of dexamethasone), and one the next day, 60 min before the onset of stress (placebo or 80 mg of propranolol). A physician was on call each day while the drugs were active in case of serious side effects. No adverse events occurred in any participant.

### Testing paradigm

**Fig. 1:**
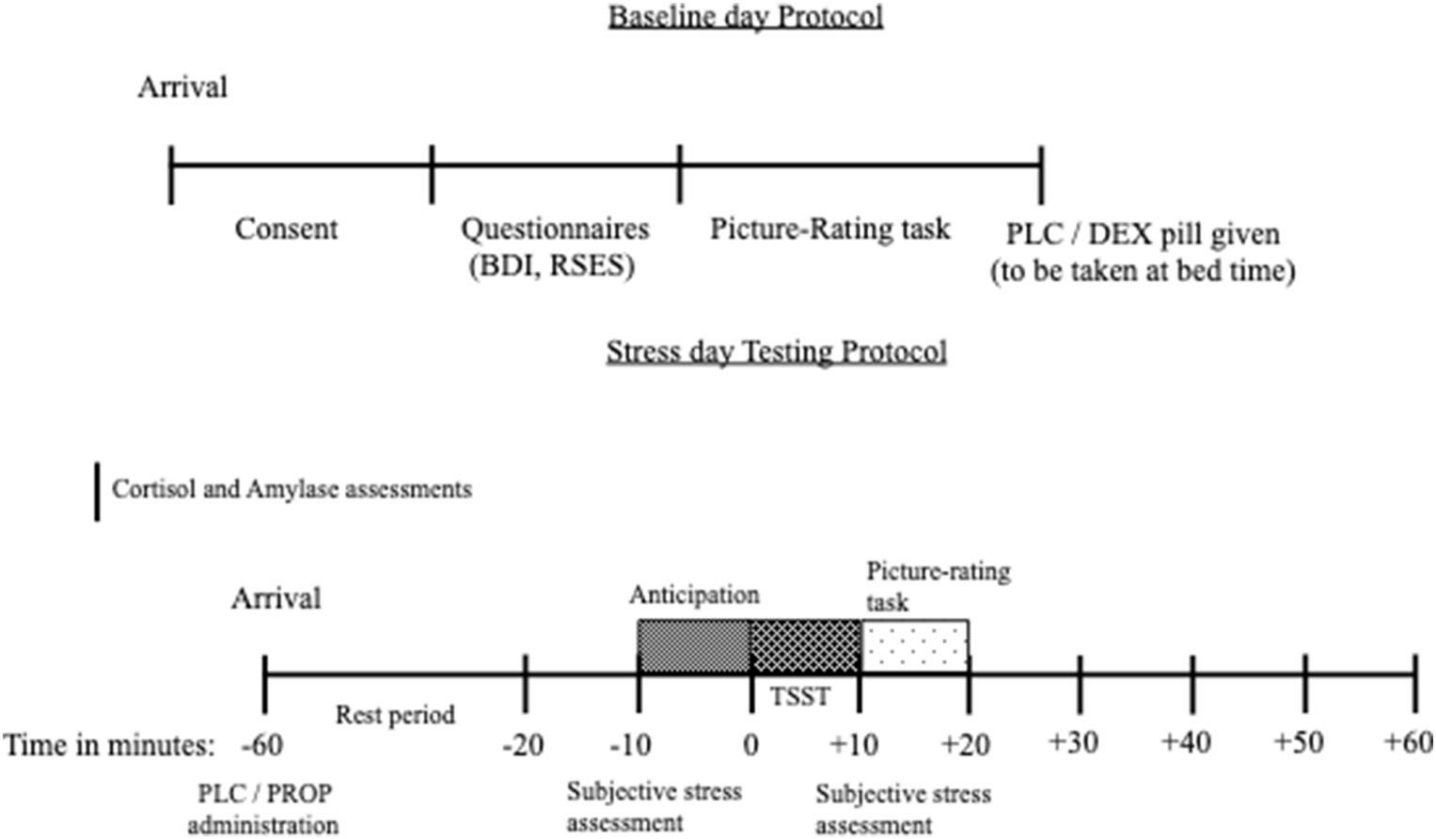
Timeline of the baseline and testing day protocols. PLC, placebo; DEX, dexamethasone; PROP, propranolol

Testing occurred on two consecutive mornings in our laboratory. On day 1, participants completed self-report assessments of self-esteem and depression (to confirm the absence of depressive symptomatology), and then completed the picture-rating task (described below). Participants then received one pill (placebo or dexamethasone), which they were instructed to take at bedtime that night (Andrews et al. 2012). Upon returning to the laboratory the next morning, participants received one pill (placebo or propranolol) 1 h before the onset of the stressor. They were seated in a waiting room for a 60-min rest period for the propranolol to take effect (Andrews and Pruessner 2013). During the rest period, participants read magazines that had been chosen for their non-stimulating content. Following the rest period, participants were exposed to the psychosocial stressor (described below). Immediately after the stress task, participants completed the picture-rating task. Stress markers, biological, and psychological were assessed throughout the duration of testing on day 2 (Fig. 1).

### Picture-rating task

**Fig. 2:**
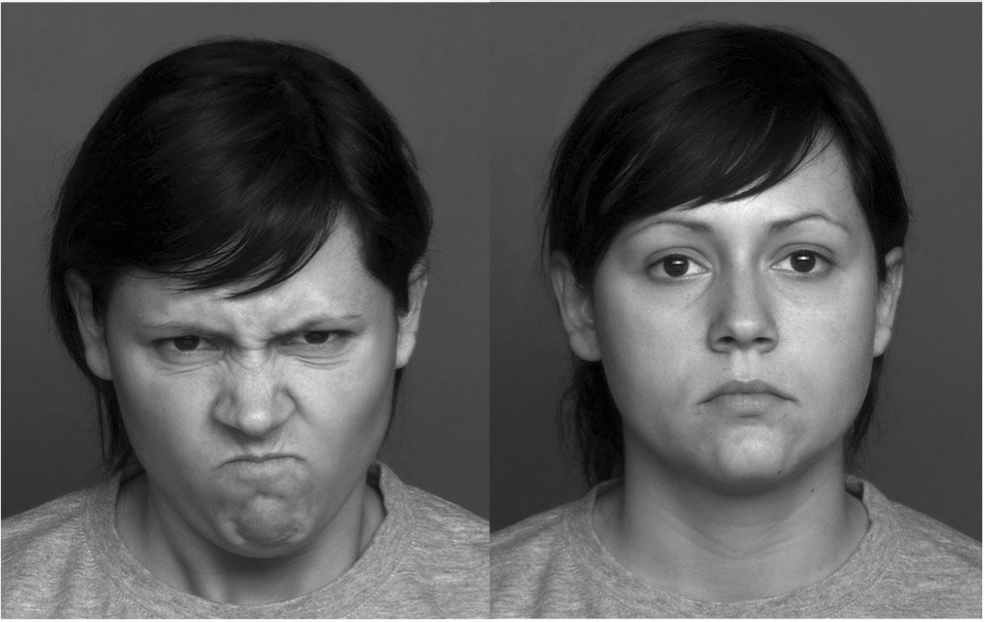
Angry and neutral faces seen during the threat assessment task at baseline (day 1), and after stress (day 2)

The picture-rating task was completed twice—day 1 (baseline), and day 2 (post-stress). On each day, participants saw 40 pictures of distinct human faces (20 angry, 20 neutral; 50% male) from the FACES database (Ebner et al. 2010), presented in randomized order (see Fig. 2 for examples of stimuli). Since the goal of the study was to assess the effects of stress on threat assessment, participants were cued to the emotionality of the faces by asking them to rate each face for how threatening they found it. Ratings were made on the number scale of a computer keyboard, ranging from 1 “not at all threatening,” to 10 (0 key on the keyboard; “extremely threatening”). Ratings 2–9 represented incrementally higher threat ratings. The task was presented on a Macintosh PowerBook laptop. Participants were seated 30 cm from the screen. Each picture was 15 × 17.5 cm in size and was presented on the screen for 10 s. Stimuli were displayed in monochrome (16 color grayscale palette) and presented using SuperCard for Mac OS X 10.4.11 (Solutions Etcetera, Pollock Pines, CA).

### Psychosocial stress paradigm

Psychosocial stress was induced using the Trier Social Stress Test (TSST) (Kirschbaum et al. 1993). The TSST consists of a 10-min anticipation phase, followed by a 10-min test phase. The test phase consists of a 5-min mock job interview, and a 5-min mental calculation task. Both tasks are performed in front of two “behavioral experts,” one male and one female. The experts are trained confederates, who remain minimally responsive and maintain neutral facial expressions, creating a tense and apprehensive atmosphere during the task. The TSST reliably produces significant elevations in cortisol and sAA (Rohleder et al. 2004; Ali and Pruessner 2012; Ali and Nater 2020).

### Stress biomarkers

To confirm that the pharmacological manipulations successfully suppressed the physiological stress responses, cortisol and sAA were analyzed from saliva samples (Sarstedt AG & Co, Nümbrecht, Germany). Cortisol levels (nmol/l) were measured using a time-resolved fluorescence immunoassay (Dressendörfer et al. 1992). Alpha-amylase (U/ml) levels were determined using the enzyme kinetic method (Engert et al. 2011). Biomarkers were collected at 9 time-points, in 10-min intervals throughout the experiment.

### Psychological assessment

Participants completed self-report measures of subjective stress, depression, and self-esteem. Subjective stress was measured using a 10-point visual-analogue-scale (VAS) (Gift 1989); asking “How stressed do you feel right now?” Responses ranged from “not at all” to “extremely.” Beck-Depression-Inventory-II (BDI) (Beck et al. 1996) was administered to confirm the absence of depressive symptomatology. Rosenberg Self-Esteem Scale (RSES) (Rosenberg 1965) was administered to assess overall feelings of self-worth and self-acceptance.

### Statistical analyses

A two-way (drug × sex) MANOVA was conducted with age, RSES, and BDI as dependent variables to ensure that participants in the different drug conditions did not differ on these variables.

Areas under the curve increase (AUCi) (Pruessner et al. 2003) were computed for *z* scores of cortisol and sAA to assess overall changes in stress biomarkers over time. *Z* scores allowed us to compare the stress systems using identical units for each system. To ensure that the pharmacological blockade successfully suppressed biomarker responses during stress, MANOVAs were conducted with AUCis of cortisol and sAA as dependent variables, and drug condition (PLC, DEX, PROP, and DP) as the independent variable.

To examine the effect of stress on threat assessment, a change score (stress– baseline) was computed for every participant’s rating of each face stimulus. A positive score indicated an increase, and a negative score indicated a decrease in sensitivity to threat for each face after stress. A linear mixed effect model (LMEM) (Holmes Finch et al. 2016) was computed to examine the relationship between threat assessment, emotion, and drug condition. Sex (male = 0), emotion (neutral = 0), drug condition (PLC = 0), and the emotion x drug interaction term were entered as fixed-effects and the intercept for subjects was added as a random-effect (Barr et al. 2013; Barr 2013). Based on the recommendation by Barr et al. (2013), *p* values were obtained by likelihood ratio tests comparing the final-model against the model that was identical in all respects except the fixed-effect in question. Confidence intervals were bootstrapped. All analyses were computed using Team, R Core (2013). The lme4 package was used for LMEM analyses (Bates et al. 2012). Multiple imputation analyses were computed for missing values (5.7% missing) using the mice package (van Buuren and Groothuis-Oudshoorn 2011).

## Results

Three participants were excluded on account of unsuccessful pharmacological suppression (values > 3 std. dev. above mean), and three were excluded for not following instructions (they rated every picture on both days with the exact same rating). Final analyses were conducted with 155 participants in the following drug conditions, PLC (*n* = 41), DEX (*n* = 39), PROP (*n* = 36), and DP (*n* = 39).

### Demographics

MANOVAs revealed no significant effects of sex or drug condition on age, RSES, and BDI (all *F*s < .80, *p*s > .30), indicating that the groups were comparable on these factors.

### Stress biomarkers—manipulation check

The MANOVA revealed a significant main effect of drug on AUCi for cortisol, *F*(3, 151) = 15.12, *p* < 0.001, *η*^*2*^ *=* 0.23; and AUCi for sAA, *F*(3, 151) = 13.87, *p* < 0.001, *η*^*2*^ *=* 0.22, indicating that dexamethasone and propranolol successfully suppressed the physiological stress responses. Post hoc tests revealed that compared with PLC, cortisol levels were significantly lower in DEX (*p* < 0.001) and DP (*p* < 0.001) conditions, and sAA levels were significantly lower in PROP (*p* < 0.001) and DP (*p* < 0.001) conditions (Table 1, supplemental Fig. S1).

**Table 1:** Means of AUCis of cortisol and salivary alpha amylase within each drug condition

	PLC *n* = 41	PROP *n* = 40	DEX *n* = 40	DP *n* = 40
Cortisol	0.24 (1.01)	0.66 (1.44)	− 0.49 (0.13)	− 0.39 (0.30)
Alpha amylase	0.37 (1.08)	− 0.49 (0.43)	0.53 (1.28)	− 0.44 (0.36)

Mean and standard deviations (in parentheses) for area under the curve increase (AUCis) of cortisol and alpha amylase across drug conditions. *PLC* placebo condition (no suppression), *PROP* propranolol (SNS suppression), *DEX* dexamethasone (HPA suppression), *DP* double suppression with dexamethasone and propranolol

### Threat assessment

Table 2 lists the mean and standard deviations of threat ratings for every drug condition on each day. The LMEM investigating changes in threat assessment, predicted by drug, emotion, and drug × emotion interaction as fixed-effects, and subject as the random-effect, revealed significant drug × emotion interactions for the comparisons between PLC and each drug condition: PLC and PROP, *b* = −0.68 (SE = 0.13; 95% − CI[− 0.97, − 0.41]), *t*(6041) = − 5.13, *p* < 0.001; PLC and DEX, *b* = − 0.49 (SE = 0.13; 95% − CI[− 0.76, − 0.23]), *t*(6041) = − 3.77, *p* < 0.001; and PLC and DP, *b* = − 0.56 (SE = 0.13; 95% − CI[− 0.82, − 0.31]), *t*(6041) = − 4.33, *p* < 0.001. Adding sex to the model did not significantly improve model-fit (model with sex, AIC = 25,412 vs. model without sex, AIC = 25,410, *p* = 0.81); therefore, the final model did not include sex. The final LMEM that included the drug x emotion interaction term (AIC = 25,410) was significantly better than the non-interaction model (AIC = 25,435; *χ*^2^(3) = 31.57, *p* < 0.001).

**Table 2:** Mean threat assessments for angry and neutral faces

		PLC	PROP	DEX	DP
Angry	Day 1	4.91 (2.58)	5.25 (2.72)	5.49 (2.59)	5.18 (2.41)
	Day 2	4.70 (2.62)	5.39 (2.70)	5.33 (2.50)	5.27 (2.48)
	*Change*	*− 0.21 (2.16)*	*0.14 (2.03)*	*− 0.16 (1.94)*	*0.09 (2.05)*
Neutral	Day 1	3.00 (2.41)	2.53 (1.90)	2.39 (2.00)	2.97 (2.05)
	Day 2	3.27 (2.50)	2.48 (1.88)	2.23 (1.76)	2.99 (2.08)
	*Change*	*0.27 (1.79)*	*− 0.05 (1.51)*	*− 0.16 (2.01)*	*0.02 (1.75)*

Mean and standard deviations (in parentheses) for threat assessment across conditions and stimuli. Day 1, no stress day; Day 2, post stress induction. The italicized values reflect the difference between day 2 values and day 1 values

Simple-slope analyses revealed that within the PLC condition, there was a significant and unpredicted, increase in threat assessment of neutral faces, compared with angry, *b* = 0.49, *t*(6041) = 5.37, *p* < 0.001.

Analyses further revealed a significant difference between PLC and PROP, with higher threat assessment for angry faces, *b* = 0.36, *t*(225.44) = 2.29, *p* = 0.02, and lower threat assessment for neutral faces, *b* = − 0.32, *t*(225.44) = −2.08, *p* = 0.03, in PROP compared to PLC. Within PROP, there was a significant increase (after stress) in threat assessment for angry faces, compared to neutral, *b* = −0.19, *t*(6041) = − 2.00, *p* = 0.04.

There were no differences between PLC and DEX on threat assessment for angry faces *b* = 0.05, *t*(225.44) = 0.33, *p* = 0.74; however, there was a significant difference for neutral faces, with lower threat assessment in DEX, *b* = − 0.44, *t*(225.44) = −2.88, *p* < 0.01. Within DEX, there was no significant difference in change in threat assessment between angry and neutral faces, *b* = − 0.002, *t*(6041) = − 0.03, *p* = 0.98.

**Fig. 3:**
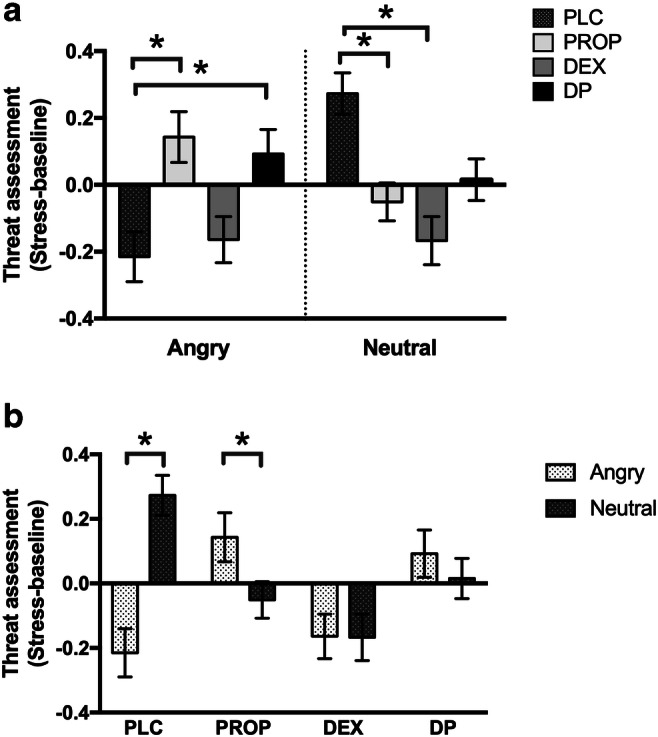
Mean (+ SEM) changes (from baseline) in threat assessment in **a**) angry and neutral face stimuli, in each pharmacological suppression condition, compared with the PLC group. **b**) angry and neutral face stimuli within each drug condition. PLC = placebo; PROP = propranolol; DEX = dexamethasone; DP = dexamethasone-propranolol

Finally, a significant difference was observed between PLC and DP, with higher threat assessment for angry faces in DP, *b* = 0.31, *t*(225.44) = 2.01, *p* = 0.04. There was no significant difference between drug conditions on threat assessment for neutral faces, *b* = − 0.26, *t*(225.44) = −1.69, *p* = 0.09. Within DP, there was no difference in threat assessment between angry and neutral faces, *b* = − 0.08, *t*(6041) = −0.83, *p* = 0.41. See Fig. 3.

### Exploratory analyses

**Fig. 4:**
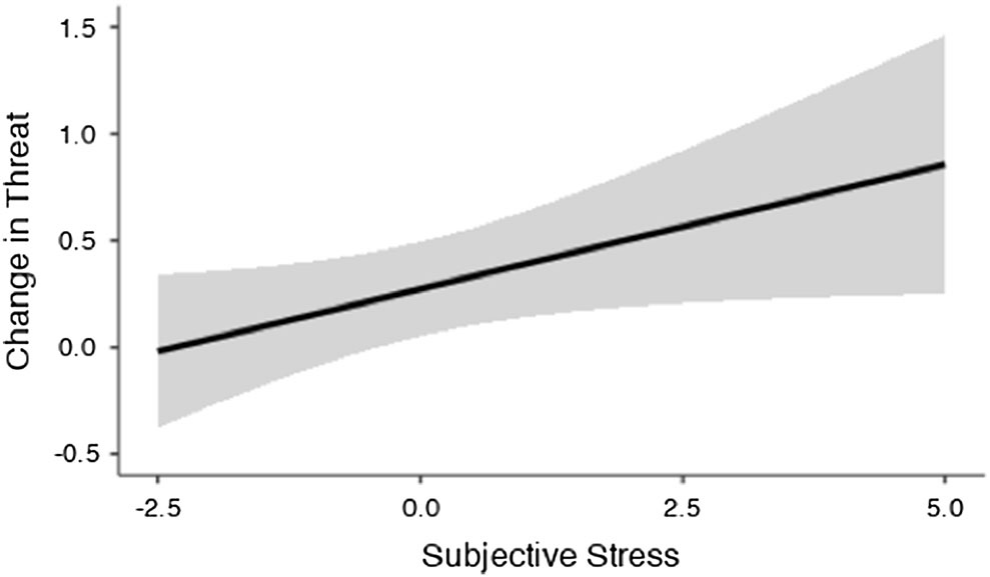
Linear relationship between subjective response to stress and change in threat assessment for neutral faces in the Placebo (PLC) condition

Since the increase in threat ratings for neutral faces in the PLC condition was contrary to our original prediction, we conducted a repeated measures MANOVA (day × emotion) to investigate whether exposure to stress (via the TSST) on day 2 was associated with differential threat assessments for angry and neutral stimuli. The results revealed a significant day × emotion interaction, *F*(1, 3239) = 10.50, *p <* 0.001. Post hoc tests revealed that compared to baseline, stress on day 2 was associated with a significant increase in threat assessment for neutral faces (*p* < 0.01), and a significant decrease in threat assessment for angry faces (*p* = 0.05). We hypothesize that this might reflect participants’ subjective experience of being evaluated by the neutral-faced experimenters during the TSST. An exploratory LMEM was conducted to investigate if the change in threat assessment for neutral faces would be associated with stress-induced increase in subjective stress (delta increase in VAS responses to stress). Subjective stress was added as the fixed-effect and subject as the random-effect. The results revealed a significant simple effect of subjective stress, *b* = 0.12 (SE = 0.05; 95% -CI[0.007, 0.23]), *t*(39) = 2.09, *p* = 0.04, indicating that a greater subjective stress response to the TSST was associated with increased threat assessment for neutral faces (from baseline) (Fig. 4). Collapsing across drug conditions, subjective stress was marginally correlated with increased threat assessment for neutral faces (*r* = 0.14, *p* = 0.06), but not angry (*r* = 0.02, *p* = 0.728) indicating that the subjective experience of stress might be more strongly linked to how threatening neutral faces were experienced after stress.

## Discussion

To examine the differential effects of physiological and psychological stress responses on threat assessment, in the context of acute stress, we used pharmacological challenges to suppress the SNS and HPA axis activity, individually and jointly. Our results suggest that functional dysregulations of the stress systems have system specific effects on threat assessment, as evidenced by the differential effects observed in our four groups.

First, we find that in the PLC condition, participants showed increased threat ratings for neutral faces after stress; a finding that seemingly contradicts our original hypothesis, and previous studies, such as for example by Dandeneau et al. (2007) who demonstrated enhanced threat processing and memory for emotional material following psychosocial stress induced using the Montreal Imaging Stress Task. In the context of our experimental paradigm, acute stress is induced by two confederates (TSST; (Kirschbaum et al. 1993), one male and one female, who maintain a deliberately neutral demeanor towards the participant to decrease social feedback, and to induce perceived social-evaluation and threat to one’s “social self” (Dickerson and Kemeny 2004). The increase in threat assessment for neutral faces may have resulted from the fact that participants completed the threat-rating task immediately after stress induction via the TSST. Given this context, we speculate that the TSST may have primed our participants to experience neutral faces as threatening, and subsequently resulted in greater emotional reaction towards the neutral stimuli during the task. This interpretation is supported by studies examining the effect of social stress on emotion processing of faces. For example, Schwarz et al. (2013) found that when neutral faces were presented in a negative self-evaluative context, they were rated as more negative and more arousing, compared with neutral faces presented in other-related contexts. Other studies have similarly demonstrated that acute stress alters how facial emotions are processed in healthy individuals (Chen et al. 2014; Daudelin-Peltier et al. 2017). Recently, Grupe et al. (2018) demonstrated that neural responses to neutral facial stimuli were differentially modulated based on the valence (negative or positive) of the faces presented immediately prior, with greater amygdalar activations for neutral faces that were presented after negative faces. Considering our results in light of this literature, we surmise that evaluating previously seen information in an altered emotional state, i.e., under stress, may have transformed the previously neutral stimuli into more emotionally salient, resulting in the observed change in threat ratings for neutral faces. This was further supported by exploratory analyses demonstrating that higher subjective stress responses to the TSST positively predicted the increase in threat assessment for neutral faces. Importantly, while global threat ratings for angry faces remained higher than for neutral, it is the change from baseline that reflected this shift in threat assessment.

Second, following SNS suppression (PROP condition), compared to placebo, acute stress resulted in decreased threat assessment for neutral faces, supporting the hypothesized role of SNS in immediate threat responses. Previous studies have implicated the noradrenergic system in increasing attentional and orienting responses to threat (Aston-Jones et al. 2000), and in attenuating responses to distracting or “noisy” stimuli (Coull 1994). Animal studies have further shown that blocking NA in rats (via LC lesions, or with propranolol) results in decreased startle responses (Walker and Davis 2002), and reduced reactivity to aversive stimuli, stemming from the inability to filter out task-irrelevant information (Mason and Iversen 1975). Mechanistically, NA is implicated in mediating amygdalar activity during emotional information processing. Specifically, emotionally salient stimuli induce increased amygdalar activation, and importantly, this activation is NA dependent (Strange et al. 2003; van Stegeren et al. 2005). Thus, suppressing NA might result in reduced amygdalar activation in response to threat, with consequential effects on reactivity to threatening stimuli. Our findings are in line with this interpretation and demonstrate that pharmacologically suppressing the SNS response was associated with reduced threat ratings for task-relevant neutral faces. In conjunction, we found increased threat assessment for angry faces in this condition. Previous studies have demonstrated that we have an adaptive predisposition to attend to emotional faces, as evidenced by autonomic and amygladar responses (Öhman 2002). Likewise, depleted and/or excessive NA levels have been shown to have impairing effects on reflective, prefrontal driven functions, resulting in rapid emotional, or habitual, and reflexive behaviors (Oei et al. 2010). Thus, it could be that in the absence of a sympathetically mediated response to help direct attention to threat, participants in the PROP condition responded with habitual and predisposed emotional responses to angry stimuli.

Third, following HPA-axis manipulation (DEX condition), compared to PLC, there was a significant reduction in threat assessment for neutral faces, and no significant effect for angry faces. These results suggest that manipulating the cortisol response may have altered the context-dependent (i.e., TSST-related) sensitivity to threat, resulting in a significant reduction in threat assessment of neutral faces. Importantly, this effect was confined to neutral faces, given that there was no effect on angry faces. The HPA axis has been known to modulate contextual responses to threat. Cortisol administration studies have demonstrated its role in enhancing goal-relevant emotional information processing (Putman and Roelofs 2011), suggesting that the inability to mount a cortisol response to stress should result in detrimental outcomes when assessing the saliency of contextually relevant emotional information. Our results support this and demonstrate that following HPA axis suppression, there was a significant reduction in threat assessment for emotionally salient/threatening stimuli (i.e., neutral faces).

Lastly, jointly suppressing SNS and HPA responses to stress (DP condition) resulted in increased threat ratings for angry faces compared to PLC, and no significant differences between drug conditions for neutral. This finding extends our recent work, in a subset of participants from this project (Ali et al. 2017) where pharmacological suppression of both systems in conjunction was associated with increased mood dysregulation and psychological stress, with no differences between PLC and DP on these measures. Our results here provide further support for the argument that the cognitive appraisal of stress may, under certain circumstances, function independently of the physiological stress responses. Thus, hormonal stress responses, when activated, serve to focus attention towards elements in the environment that pose threat by suppressing non-relevant distracters, allowing one to actively cope with stress in a goal directed manner (Schwabe et al. 2010; Putman and Roelofs 2011). In the absence of these physiological responses, the system appears indiscriminately sensitive to all potential threats, resulting in increased threat assessment of both contextually relevant (neutral) and irrelevant (angry) stimuli. However, given that this study utilized angry and neutral stimuli only, whether this represents an indiscriminate attention to all stimuli, or all threatening stimuli, remains to be examined.

Our results should be considered alongside their limitations, and implications for future research. We postulate that the TSST contextually altered the emotional saliency of neutral faces. However, since we did not have a no-stress condition, we were not able to empirically test this and the interpretation of that finding is speculative Thus, future studies using these pharmacological manipulations should include a no-stress control condition to investigate this effect. Additionally, our threat assessment paradigm consisted of only angry and neutral stimuli; thus, we cannot draw conclusions about how our findings will extend to other negative or threatening (sad, fearful), or positive (happy) stimuli. Moreover, we did not find significant sex effects. This might be because female participants were tested during the luteal-phase of their menstrual cycles. Previous studies have shown important effects of menstrual phases on physiological stress responses (Kirschbaum et al. 1999), and emotional attention processing (Yamazaki and Tamura 2017). Follow-up studies should consider including women in the follicular-phase, and on oral contraceptives, to investigate how interactions between the stress systems impact threat processing in women across different menstrual phases. Additionally, we administered propranolol and dexamethasone to suppress SNS and HPA axis responses to stress, respectively. These agents effectively block sAA release by inhibiting beta-adrenergic receptor activation, and cortisol release via feedback inhibition on the pituitary (Andrews et al. 2012; Andrews and Pruessner 2013), and can safely be administered in humans (without causing serious side effects when taken together) (Ali et al. 2017). However, we recognize that the effects of propranolol and dexamethasone on the physiology of the stress systems may be more widespread and nuanced, and that both pharmacological agents may have effects that extend beyond the observed decreases in sAA and cortisol responses. In particular, since propranolol crosses the blood-brain-barrier, it may have only partially blocked the effects of peripheral or central A and NA, via effects on beta-adrenergic receptors. Follow-up studies should consider including an atenolol (a beta-blocker that acts peripherally) comparison group, to distinguish between central and peripheral SNS effects. Conversely, dexamethasone does not cross the blood-brain-barrier (de Kloet et al. 1974), and acute stimulations of the HPA axis may have resulted in central increases in CRF, which may have contributed to some of our effects. Follow-up studies should consider metyrapone (a corticosteroid synthesis inhibitor) administration to more thoroughly investigate the observed effects. Relatedly, while this study provides important behavioral evidence regarding differential effects of stress hormones on threat-reactivity, given that we used an acute dosing paradigm, we can only speculate about the functional effects of these manipulations, which may differ between acute and chronic dysregulations of the stress systems.

These limitations notwithstanding, this study represents the first attempt in humans to investigate how each stress system, independently activated, modulates social threat assessment. Our results demonstrated that intact biological stress systems adaptively support organisms during stress by focusing attention on specific stimuli that are relevant to the threat. Dysregulations of these systems have important system-specific consequences on threat evaluation, which could subsequently impact coping responses, as observed in stress-related pathologies. This represents an important area of study since there is significant evidence of dysregulated cross-talk between the stress systems in clinical (Gold and Chrousos 2002; Allwood et al. 2011), and vulnerable populations (Ali and Pruessner 2012), and biased responses to social threat are observed in these conditions (Peckham et al. 2010; Pechtel and Pizzagalli 2011). A systematic understanding of the mechanisms that contribute to these stress related detrimental effects can therefore provide us with important insights regarding underlying cognitive vulnerabilities that are associated with the development and maintenance of stress-related adverse mental health outcomes.

## Electronic supplementary material

ESM 1 (DOCX 110 kb)
