# Supplementary material for: The effects of suppressing the biological stress systems on social threat-assessment following acute stress
Source: Psychopharmacology (Berl). 2020 Jun 29;237(10):3047–56. doi: 10.1007/s00213-020-05591-z (PMC7525279; doi:10.1007/s00213-020-05591-z)
Supplement: Supplementary file 1 — (DOCX 110 kb) [file 213_2020_5591_MOESM1_ESM.docx]

**Supplemental Material for:**

**The effects of suppressing the biological stress systems on social threat-assessment following acute stress.**

Nida Ali^1,2,4^, Cory Cooperman^1,2^, Jonas P. Nitschke^1,2^, Mark W. Baldwin^1^, Jens C. Pruessner^2,3^

^1^Department of Psychology, McGill University, Montreal, Canada

^2^Faculty of Medicine, McGill Centre for Studies in Aging, McGill University, Montreal, Canada

^3^Department of Psychology, University of Konstanz, Konstanz, Germany

^4^Department of Psychology, University of Vienna, Vienna, Austria

**This document includes**:

S1. Figure depicting the biomarker responses for stress markers in each drug condition

**Figure S1:** Mean area under the curve increase (AUCi) values (+ SEM) for **A)** cortisol and **B)** alpha-amylase responses to stress, in the Placebo (PLC), Propranolol (PROP), Dexamethasone (DEX), and Dexamethasone- Propranolol (DP) conditions.
